# Comparison of Prevalence and Risk Factors of Acute Coronary Syndrome in Patients with Different Ethnicity: A Cross-sectional Study

**DOI:** 10.4314/ejhs.v31i5.13

**Published:** 2021-09

**Authors:** Homeira Khoddam, Zobeide Alemi, Mahnaz Modanloo

**Affiliations:** 1 Nursing Research Center, Golestan University of Medical Sciences, Gorgan, Iran; 2 Student Research Committee, School of Nursing and Midwifery, Golestan University of Medical Sciences, Gorgan, Iran

**Keywords:** Acute Coronary Syndrome, Risk factors, Ethnic groups, Heart Diseases, Iran

## Abstract

**Background:**

Although the main risk factors of acute coronary syndrome (ACS) have been previously identified, there is not yet strong and consistent evidence about the ethnical differences of these risk factors. The aim of this study was to identify and compare the distribution of risk factors of ACS among two ethnic groups in northern Iran.

**Methods:**

This cross-sectional study was done on a total of 250 patients (100 Fars and 150 Turkmen ethnics) with ACS admitted in coronary care units (CCU) of medical centers in Gonbad-e Kavus, a city in the Northeast of Iran. The demographic characteristics, clinical parameters and anthropometric indices of patients in two ethnic groups were recorded. In addition, Beacke's questionnaire and Cohen's scale were used to evaluate and compare the patients' level of physical activity and perceived stress, respectively.

**Results:**

The mean age of the patients was 60.9±11.9 years and they were mostly males (54.8%) and married (84.8%). Findings showed that the prevalence of myocardial infarction in Fars patients was significantly higher than Turkmens (24% versus 15.3%; P=0.04). In addition, there was a significant difference in terms of the history of using opium (P=0.07) and opium sap (P=0.03), socioeconomic status (P=0.009), the place of residence (P=0.001) and type of health insurance services (P=0.001) between two groups. However, the clinical parameters and anthropometric indices and the level of physical activity and perceived stress were not significantly different between two groups (P>0.05).

**Conclusion:**

This study showed a significant difference in the prevalence and risk factor of ACS in patients with different ethnicity in northern Iran. This finding points to the importance of paying attention to the ethnicity-based difference in ACS prevalence and risk factors, especially in patients who are at high to intermediate risk for ACS, such as Turkmens.

## Introduction

Cardiovascular Diseases (CVD), including acute coronary syndrome (ACS), have been the cause of 17.9 million deaths in 2018, which approximately accounts for about 23% of deaths worldwide (1–2). It has been previously shown that there is a significant difference in the mortality rate caused by CVDs in high and low- and middle-income countries (LMICs)(3).The Eastern Mediterranean region (EMR) consist of more than 20 LMICs, which is predicted to have a significant increases in cardiovascular mortality over the next decades (4). It has been reported about 50% of CVDs' mortality and 80% of global burden of the disease occurred in this region (5). Iran may have the highest burden of CVD in the EMR(2).

According to previous studies, not only the prevalence but the diversity and the number of risk factors for ACS are increasing in the Iranian population (5). In addition, some studies confirmed the role of some modifiable risk factors such as smoking, inactivity, stress, increased cholesterol level, overweight and obesity and a history of hypertension in occurrence of ACS among Iranian population (5–8). Despite of the growing evidence about the risk factors, the differences in incidence and complication of CVDs in different societies are still not justified. Therefore, assessing the role of social determinants of health such as education, health literacy, access to health care, habits, and psychosocial factors are reasonable (9). Previous studies confirmed the role of ethnicity as an important social determinant of health and its association with various diseases, including coronary heart disease. These studies have shown that ethnic minorities are disproportionately at higher risk for heart disease (9–12). However, there is a little evidence to justify differences in the distribution and impact of the risk factors among different ethnic groups. This may be due to the small number of studies reflecting ethnic and cultural diversity and its contribution to the risk of coronary heart disease (9, 10, 13). Despite several population-based studies on CVDs in Iran (2, 14), a few number of them have focused on the diversities between different ethnic groups (8, 15). Indeed, in spite of the existence of several ethnic groups in Iran that account for 30% of the total population (16), the evidence about the potential role of ethnical variations in the distribution and severity of CVDs' risk factors is insufficient (15). It is believed that, conducting observational and interventional studies in LMICs, focusing on different race/ethnic groups, will improve the body of knowledge for managing CVDs globally (3). In addition, assessing the history of patients with CVDs may provide an invaluable evidence for developing preventative programs in both healthy and vulnerable individuals (7). By considering the scarce evidence about CVDs in Turkmen ethnic group which mainly living in Northeastern region of Iran (16), we aimed to identify and compare the distribution of risk factors of ACS in the Fars and Turkmen patients admitted to the coronary care units (CCUs).

## Methods

This cross-sectional study was conducted from May to November in 2018. The study population were patients with ACS from two ethnic groups, Fars and Turkmen, admitted to CCUs of medical centers in Gonbad-e Kavus, a city in the Northeast of Iran.

The participants were selected by a purposive sampling method based on the eligibility criteria. The inclusion criteria were belonging to the Fars or Turkmen ethnic group and hospitalized as a case of the ACS. The exclusion criteria were inability to communicate verbally and having serious complications such as severe arrhythmias or cardiogenic shock. Patients' demographic characteristics (age, gender, marital status, education level, occupation, the place of residence and socioeconomic status of the patients), clinical characteristics (family history of heart disease, history of hypertension and hyperlipidemia, diabetes and drug abuse, blood pressure and blood lipid and sugar profiles at admission and medications used) and anthropometric indices (weight, height, body mass index and waist and hip circumference) were assessed and recorded. Socioeconomic status was classified based on occupation, income and educational status of patients (17). Socioeconomic status determined by combining the patients' occupational status [scores 1 to 3], education level [scores1 to 3] and place of residence [scores 1 to 2] and rated as low (3), middle (4–6) and high (7–8).

The weight of the participants was measured at the time of admission, using similar digital scale (Seca 707; range 0.1–150 kg) for everyone, while the patients dressed the hospital clothes. Their height measured using one wall-fixed tape, while standing without shoes against the wall. The Body mass index (BMI) was calculated using the following formula: weight (kg)/ [height (m)]^2^. All para-clinical parameters were recorded based on the patients' laboratory reports which had been done by similar methods and kits.

To evaluate the levels of patients' physical activity and perceived stress, the Baecke's questionnaire (18) and Cohen's scale (19) were used, respectively. The Baecke physical activity questionnaire consists of three indicators of occupational activities, sport and recreational activities. The first section includes eight questions on various body positions during working with a score of 1 to 5. The overall score is obtained by dividing the total score by 8. The second section includes four questions on sports activities, and the overall score of this index is therefore obtained by dividing the sum of scores by 4. The third section includes four questions on the recreational activity. A score of 1 to 5 is assigned to each question and the overall score of this section is obtained by dividing the acquired score by 4. The sum of scores acquired for the above three indicators determines the level of patients' physical activity. A higher score, indeed, shows a higher physical activity level (18).

The Perceived Stress Scale (PSS) developed by Cohen *et al.* includes 14 items with a 5-point Likert scale (none, low, medium, high and very high) ranging from 0 to 4. The sum of scores shows the level of stress perceived by an individual (20–22). Based on the achieved scores (0–56), we categorized levels of perceived stress to low, medium and high, if scores were between 0–18, 18–36 and above 36, respectively (Tertile). In this study the reliability of Baecke's questionnaire and Cohen's scale calculated using the internal consistency method. The Cronbach's alpha coefficients were 0.76 and 0.71, respectively.

**Sample size estimation**: The sample size was estimated based on a pilot study on 20 eligible patients. The sample size was calculated using the formula for comparing two independent means, for each risk factors and then based on the higher sample size for HDL, A total of 230 participants were counted for the study. Taking into account a 10% loss, a final sample size of 250 was estimated (SD Turkmen=13.2, SD Fars= 8.7, α=0.05, β=0.1). At the end, according to the ratio of people with Turkmen and Fars ethnicities in the community, the sample size in each group was estimated 150 and 100, respectively. The sample size assigned to each medical centers was determined based on the bed occupancy rate of the CCUs.

**Ethical consideration**: Data collection began after obtaining the approval of the ethics committee of Golestan University of Medical Sciences and a written informed consent from the participants.

**Statistical analysis**: The normality of the data was assessed using Shapiro-Wilks test. Data were analyzed using independent t test, chi-square, Fisher's exact tests and Mann-Whitney test in SPSS software (version 18, SPSS Inc., Chicago, IL, USA). For all statistical tests, P value <0.05 was considered as statistically significant.

## Results

According to the result, patients' mean± standard deviation (SD) of age was 60.9±11.9 year, majority of them were male (54.8%), married (84.8%) and had 4–7 children (52.4%). There were not any significant differences between two groups in terms of these characteristics. In addition, 66% of patients had a low level of education and mostly were unemployed (48.8%). Majority of participants had a part time job (61.6%) and had not a permanent income (57.2%). The level of socio-economic status of them (45.6%) was low. Analysis of the demographic characteristics revealed a significant difference between the Fars and Turkmen ethnic groups in terms of the place of residence (P=0.001), education level (P=0.01), occupation (P=0.001), Job status for employed persons (P=0.001), income (P=0.001) and socioeconomic status (P=0.001) (Table 1).

**Table 1:** The comparison of demographic characteristics of patients in Fars and Turkmen ethnic groups

Demographic characteristics		Ethnic group		Total N (%)	P-Value

		Fars N (%)	Turkmen N (%)		
Gender	Female	43 (43)	70 (46.7)	113 (45.2)	0.56
	Male	57 (57)	80 (53.3)	137 (54.8)	
Marital status	Married	83 (83)	129 (86)	212 (84.8)	0.51
	Widow or Divorced	17 (17)	21 (14)	38 (15.2)	
Number of	0–3	34 (34)	41 (26.8)	75 (29.4)	0.059
children	4–7	55 (55)	75 (50.3)	130 (52.4)	
	>7	11 (11)	34 (22.8)	45 (18.1)	
Place of	Urban	88 (88)	58 (38.7)	146 (58.4)	0.001
residence	Rural	12 (12)	92 (61.3)	104 (41.6)	
Education	Low literacy/ Illiterate	58 (58)	107 (71.3)	165 (66)	0.01
	Diploma/ less	26 (26)	35 (23.3)	61 (24.4)	
	Academic	18 (18)	8 (5.3)	24 (9.6)	
Occupation	Unemployed	41 (41)	81 (54)	122 (48.8)	0.001
	Employed	28 (28)	58 (38.7)	86 (34.4)	
	Retired	31 (31)	11 (7.3)	42 (16.8)	
Job status for	Full time	15 (53.6)	18 (31)	33 (38.4)	0.001
employed persons	Part time	13 (46.4)	40 (69)	53 (61.6)	
Income Status	Permanent	56 (56)	51 (34)	107 (42.8)	0.001
	Temporary	44 (44)	99 (66)	143 (57.2)	
Socio-Economic	Low	36 (36)	78 (52)	114 (45.6)	0.009
level	Middle	47 (47)	57 (38)	104 (41.6)	
	High	17 (17)	15 (10)	32 (12.8)	

The results on type of insurance services coverage indicated a higher percentage of Fars patients in comparison with Turkmens used private complementary insurance services (26% vs 14%). In addition, the rate of social welfare services coverage in Fars patients was more than that in Turkmens (38% vs. 25.3%), while most Turkmen patients were covered by rural insurance (44% vs. 14%). All these differences between the two ethnic groups was statistically significant (P<0.001).

According to the findings, the mean of weight, height, BMI, waist and hip circumferences in Turkmen patients were more than the Fars one, although these differences were not statistically significant. Similarly, despite the higher waist-to-hip ratio in Fars than in Turkmen, this difference was not statistically significant (Table 2).

**Table 2:** The comparison of Anthropometric indices of patients in Fars and Turkmen ethnic groups

Anthropometric indices	Ethnic group		P-value

	Fars Mean (SD)	Turkmen Mean (SD)	
Weight (kg)	57.6 (15.6)	77.1(14.5)	0.28*
Height (cm)	165.8 (8.1)	167.4(9.2)	0.22**
BMI	27.2 (4.8)	27.5(4.9)	0.83**
Waist Circumference (cm)	100.2(12.6)	101.6(12.7)	0.92*
Hip Circumference (cm)	99.8 (11.8)	101.9(12)	0.36**
Waist-Hip Ratio(Female)	1 (0.16)	1 (0.08)	0.69**
Waist-Hip Ratio (Male)	1(0.07)	1(0.07)	0.99**

* T- Test

** Mann-Whitney u test

Despite the different mean and SD of systolic and diastolic blood pressures, fasting blood sugar and blood lipid profiles of the Fars and Turkmen patients at the time of admission, the observed differences in these parameters were not statistically significant (Table 3).

**Table 3:** The comparison of clinical parameters of patients in Fars and Turkmen ethnic groups

Clinical parameters	Ethnic group		P-value

	Fars Mean (SD)	Turkmen Mean (SD)	
Systolic BP (mmHg)	125.6 (20.4)	121.5 (19.3)	0.46**
Diastolic BP (mmHg)	77.2 (13.9)	74.5(14.7)	0.90**
Blood Glucose (mg/dl)	172(113.5)	155(91.8)	0.53**
Fasting Blood Sugar (mg/dl)	152.2 (83.5)	138.5 (68.3)	0.30**
Triglyceride (mg/dl)	134.3(66.8)	134.9 (105.8)	0.28**
Cholesterol (mg/dl)	174.1(47.2)	172.3(45.4)	0.35*
LDL (mg/dl)	108 (38.8)	104(41.1)	0.59**
HDL (mg/dl)	38.6 (10)	42 (9)	0.22**

* T- Test

** Mann-Whitney u test

Moreover, the difference observed among the Fars and Turkmen patients in terms of the history of hyperlipidemia (35% vs 30.8%), diabetes (26% vs 30%) and hypertension (57% vs 62%) was not statistically significant.

According to the results, there was a significant difference between two ethnic groups in terms of history of using opium (P=0.07) and opium sap (P=0.03). It means that the history of opium and opium sap use was higher in the Turkmen patients (22% and 13.3%) in compared with Fars patients (13% and 10%). However, no significant difference was found between two groups in terms of smoking history (almost 11% in both groups; P=0.81).

In addition, the findings indicates a significant difference between Fars and Turkmen patients in the type of ACS (P=0.04). It means, the prevalence of myocardial infarction in Fars patients was significantly higher than Turkmen patients (24% versus 15.3%).

Analysis of data on the physical activity level of patients indicated the lower level of activity in the Turkmen patients than in the Fars patients in occupational activities, sport and recreational activities, although these differences were not statistically significant (Table 4).

**Table 4:** The comparison of physical activity level of patients in Fars and Turkmen ethnic groups

Physical activity indices	Ethnic group		P-value

	Fars Mean (SD)	Turkmen Mean (SD)	
Occupational activities	2.8 (0.5)	2.8 (0.4)	0.77**
Sport	2.2(0.7)	2.1 (0.6)	0.07**
Recreational activities	1.8 (0.7)	1.7(0.5)	0.64**
Total score	6.8(1.21)	6.6 (1.2)	0.19**

** Mann-Whitney U test

The study findings regarding the perceived stress level in Fars and Turkmen patients showed that the both ethnicities perceived moderate level of stress. However, the observed differences in the mean and SD of scores in Fars (28.1±6) and Turkmen (26.6±6) patients were not statistically significant (P=0.34).

## Discussion

The results of this study indicated a significant difference between Fars and Turkmen ethnic group patients in some risk factors of ACS. Based on the results, there was a statistical difference in terms of using opium and opium sap among Fars and Turkmen patients. This is consistent with the results of Zakerkish *et al.* who found a signifciant difference among patients in Kerman and Arab patients in Ahwaz in terms of using opium and opium- contained substances (23). The higher frequency of opioid use in the Turkmen ethnic group can be related to more avaiability of opioids due to the neighburing with opioid-producing countries and a common belief in Turkmens regading the therapeutic effects of opium.

As results shows, the difference in the education level, occupational status and income of the Fars and Turkmen patients was statistically significant. This finding is consistent with Graham *et al.* study on various American races and ethnic groups with ACS. According to their results, black and Spanish patients had lower education and income levels than non-Spanish white patients (24). The mentioned characteristics are part of a set of social determinants of this ethnic group which reflect the socioeconomic status of them and determine their sustainability for cardiovascular disease. As finding showed, the socioeconomic status of Turkmen patients was significantly lower than Fars patients. This is consistent with other studies which found the socioeconomic status as a related factor of disparities among racial and ethnic minorities in developing CVDs (24, 25). In addition, literature confirmed the role of socioeconomic status and quality of life in dealing with the risk factors of heart diseases (26, 27).

Based on the findings, the type and coverage of insurance services for Turkmen patients in comparison with Fars patients was more limited. This findings confirms with numerous studies assessing the insurance coverage and socioeconomic levels of ethnic/racial groups and their relation with access to health services (24, 27–30). The higher rate of settlement in the rural area and the lack of permanent jobs and a fixed income in the Turkmen population will justify this difference. No significant difference was found in this study between the two ethnic groups in terms of risk factors such as hypertension, diabetes, hyperlipidemia and obesity. However, Abbasi *et al.*reported those as risk factors among the Iranian and also Pakistani ethnic groups (27, 28). The differences between these studies may originate from the applied data gathering methods. We used a self-report questionnaire that may affect by patients' recall and awareness. Another reason for different findings may be due to the distinctive features of research design and studied ethnic groups.

Based on the findings, the prevalence of myocardial infarction in Fars patients was significantly more than Turkmen patients. This finding is inconsistent with Graham's study. He showed that the minorities with ACS have a higher mortality rate and are more susceptible to myocardial infarction (30). This inconsistency may due to the underestimation of prevalence of myocardial infarction in the Turkmen ethnic group. In addition, it may the result of some predisposing factors of myocardial infarction such as biologic and genetic characteristics in different ethnic groups. Although based on the previous studies, inactivity is a major risk factor for ACS and also a mediating risk factor of obesity, hyperlipidemia and hypertension in the cardiovascular diseases (25, 31), no significant difference was found in this study between the two ethnic groups in terms of physical activity level, obesity, hypertension and hyperlipidemia. This disagreement can be attributed to differences in the population under study. Most studies that reported these risk factors for heart diseases, have been conducted on healthy individuals (7). However, this study aimed at determining the differences of patients facing these risk factors in terms of ethnicity. The lack of difference in the BMI and other anthropometric indices in this study is consistent with the results of Vaghari *et al.* who found no difference in the BMI between Fars and Turkmen ethnic groups (32).

Regarding the level of perceived stress, the results of the study showed that the patients in both ethnic groups perceived the moderate level of stress, although there was not a significant difference between them. This finding is consistent with those obtained by Shahabadi *et al.* for patients with coronary artery stenosis. They reported a moderate to severe stress level in the hospitalized patients (7). It seems, living in the same community with the similar sources of stress can justify the equally level of perceived stress in studying groups. In addition, doing the study on hospitalized patient may contribute to take this result. This study has some limitations. First, the cross-sectional design of the present study limits our ability to form firm conclusions regarding causality. Second, the result of this study may not be generalizable to other different ethnicities than Turkmens who live predominantly in north of Iran or other countries with different cultures, economic, and political backgrounds.

In conclusion, the results of this study revealed a significant difference in the prevalence and risk factors for developing ACS in patients with different ethnicity in northern Iran. These findings points to the importance of paying attention to the ethnicity-based risk factors in patients who are at high to intermediate risk for ACS and highlight the need for more targeted interventions for reducing the incidence as well as burden of ACS in population.
